# The Ipsilesional Upper Limb Can Be Affected following Stroke

**DOI:** 10.1155/2013/684860

**Published:** 2013-11-26

**Authors:** Gemma H. Kitsos, Isobel J. Hubbard, Alex R. Kitsos, Mark W. Parsons

**Affiliations:** ^1^Stroke Research, Neurology Department, John Hunter Hospital, Hunter New England Local Health District, Locked Bag 1, Hunter Regional Mail Centre, NSW 2310, Australia; ^2^School of Medicine and Public Health, University of Newcastle, Callaghan, NSW 2308, Australia; ^3^Hunter Brain Injury Service, Hunter New England Local Health District, Bar Beach, NSW 2300, Australia

## Abstract

*Objective*. Neurological dysfunction commonly occurs in the upper limb contralateral to the hemisphere of the brain in which stroke occurs; however, the impact of stroke on function of the ipsilesional upper limb is not well understood. This study aims to systematically review the literature relating to the function of the ipsilesional upper limb following stroke and answer the following research question: Is the ipsilesional upper limb affected by stroke? *Data Source*. A systematic review was carried out in Medline, Embase, and PubMed. *Review Methods*. All studies investigating the ipsilesional upper limb following stroke were included and analysed for important characteristics. Outcomes were extracted and summarised. *Results*. This review captured 27 articles that met the inclusion criteria. All studies provided evidence that the ipsilesional upper limb can be affected following stroke. *Conclusion*. These findings demonstrate that clinicians should consider ipsilesional upper limb deficits in rehabilitation and address this reduced functional capacity. Furthermore, the ipsilesional upper limb should not be used as a “control” measure of recovery for the contralateral upper limb.

## 1. Introduction

Neurological dysfunction commonly occurs in the upper limb contralateral to the hemisphere of the brain in which stroke occurs; however, the effect on the ipsilesional upper limb (iUL) is poorly understood [1, 2]. Contralateral deficits increase reliance on the iUL for function and for maintaining independence [3, 4]. Recognising the impact of stroke on the iUL is an important step towards implementing effective rehabilitation and to improve our understanding of the challenges faced following stroke [5, 6].

Health professionals commonly use the iUL as a measure of reference for recover, and frequently refer to it as “nonaffected” or “unaffected” [2, 7]. For health professionals to simply presume that the iUL is not affected by stroke, as our current terminology infers, may fail to adequately recognise the contribution of a functionally important component of upper limb recovery.

In this study, the term iUL refers to the arm and hand on the same side of the body as the lesioned hemisphere. This study will systematically review the research investigating the iUL following stroke to determine if the iUL is affected or not affected by stroke. This study hypothesises that following stroke the iUL can be adversely affected.

## 2. Method

### 2.1. Search Strategy

A systematic search of the literature was carried out in October 2012 using the following databases: Medline, Embase, and PubMed. Search strategies were developed in accordance with the requirement of each database to locate studies for inclusion. The following search terms were used: stroke, upper limb, upper extremity, arm, less affected, nonaffected, and ipsilateral. An example search strategy has been included (Table 2). A further manual search was conducted from the reference lists of the “captured” studies to identify other relevant studies for inclusion.

### 2.2. Inclusion/Exclusion Criteria

**Figure 1:**
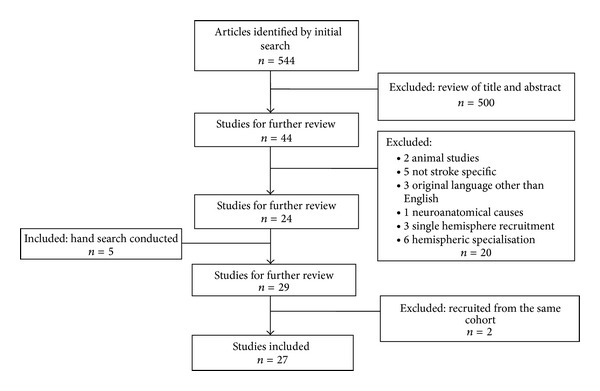
Selection of studies.

This review only included articles reporting original research that recruited adult stroke survivors. It excluded studies not initially published in English, conference publications, and those that used animal modelling. It also excluded studies which only recruited patients with a left or a right hemispheric stroke, studies which aimed to analyse the role of each hemisphere and/or the function of the iUL, and studies which explored neuroanatomical causes for iUL deficit (Figure 1).

### 2.3. Selection of Studies

From the initial search, titles and abstracts were reviewed for relevance. Studies which appeared to meet the inclusion criteria were then analysed using the full text. Once the inclusion criteria were confirmed, relevant data was then extracted by the review panel in accordance with a customised data collection form. Where discrepancies arose, the review panel reached agreement through discussion.

## 3. Results

This systematic review found that the iUL can be affected following stroke. The search captured 27 studies which assessed iUL motor and/or sensory deficits following stroke (Table 1).

The iUL was reported as affected in all of the 27 studies captured by this review. The publication dates ranged from 1971 to 2012, with eight (29.6%) studies published before the year 2000. The number of participants with stroke ranged from seven participants to 100; mean (SD) participant cohort was 33.2 (22.8) years. Participant ages ranged from 50.1 to 72.4 years; mean (SD) age was 60.7 (6.1). Isolated deficits of the iUL were not reported; contralateral upper limb deficits were present in all participants recruited to the stroke cohort across the 27 included studies.

Only Noskin et al. [1] and Spaulding et al. [8] compared a stroke cohort to normative data, whilst all the remaining studies (*n* = 25, 92.6%) compared results to age-matched healthy controls. A mixed cohort of left-handed and right-handed participants was recruited in six studies, whilst 18 studies (66.7%) recruited right-handed participants only. Hand dominance data was incomplete in the remaining three studies [2, 9, 10].

Standardised assessments were utilised in 12 (44.5%) studies to explore iUL deficits [1–8, 11–14]. Noskin et al. [1], Yelnik et al. [15], and Morris and Van Wijck [12] assessed upper limb function using the Nine Hole Peg Test (9HPT) [16], and Sunderland et al. [7], Wetter et al. [3], Jebsen et al. [14], and Spaulding et al. [8] utilised the Jebsen Hand Function Test (JHFT) [9]. Laufer et al. [4] assessed with both the 9HPT and the JHFT. The Action Research Arm Test was used by Morris and Van Wijck [12] and Nowak et al. [17]. 

A dynamometer was the most frequently used assessment tool to determine strength (*n* = 5, 18.5%) [1, 7, 10, 13, 18]. Noskin et al. [1] reported that grip strength was not significantly affected at the time points assessed: 24–48 hours, one week, three months, and one year after stroke. Sunderland et al. [7] reported that grip strength was reduced within one month of stroke (*P* < 0.001), and in a subsequent study [19] they reported that grip strength had significantly improved at six months after stroke. McCrea et al. [10] reported that 12 months after a stroke event, strength remained affected in the iUL (*P* < 0.001).

Both standardised and nonstandardised assessments were used in seven studies (25.9%) [10, 15, 17, 18, 20–22]. A further eight studies (29.6%) used only nonstandardised assessments and employed a case-control study design [23–30]. When considering the primary outcome of the studies, Brasil-Neto and De Lima [13] focused on sensory deficits, Sunderland et al. [7] investigated cognitive deficits, and the remaining studies measured motor deficits (*n* = 25, 92.6%).

When considering time after stroke, four (14.8%) studies [1, 2, 18, 20] recruited participants in the acute phase after stroke (≤one week), nine (33.4%) studies [4, 7, 11, 12, 15, 17, 21, 23, 24] recruited participants in the subacute phase after stroke, and 12 (44.5%) studies investigated a chronic stroke cohort (≥six months). Baseline assessment was unable to be determined in further two studies (7.4%) [8, 27]. Baskett et al. [21], Jung et al. [2], Noskin et al. [1], Laufer et al. [4], and De Groot-Driessen et al. [11] assessed change over multiple time points.

## 4. Discussion

This review found evidence that stroke can adversely affect the iUL. To our knowledge, this is the first systematic review of iUL performance to date. This review demonstrates that iUL deficits can be present in the acute, subacute, and chronic phases of stroke recovery. Of the 27 studies reviewed, eight were published before the year 2000 demonstrating that this is not a new concept in stroke research; however, despite current supportive evidence, it continues to be poorly recognised and understood [2, 7]. This evidence challenges the current clinical vocabulary which refers to the “nonaffected” or “unaffected” iUL [2, 7]. It also reinforces the fact that health professionals should not be using the iUL as a “control” measure for dysfunction in the contralateral upper limb.

This review has demonstrated that, as with the contralesional upper limb, there is a broad range of measures that can be used to assess iUL impairment. It provides evidence that the 9HPT [1, 4, 12, 15] and JHFT [3, 4, 7–9, 14] are sensitive to motor impairment in the iUL. Both assessments are used routinely in patients recovering from stroke, and the 9HPT has been validated for use in this cohort in a systematic literature review [31]. In contrast, grip strength of the iUL was reported to be both affected [7, 10] and unaffected [1, 18] across the acute, subacute, and chronic phases of stroke recovery. These conflicting results may be reflective of between-study differences in the participant cohort, or they may reflect the fact that this type of measure is, in fact, a crude de facto measure of corticospinal tract integrity [32].

### 4.1. Clinical Relevance

The current systematic review finds evidence of abnormal patterns of movement and strength in the iUL. These deficits can be linked to reduced functional capacity following stroke and may impact patient outcomes [1, 5, 11]. These findings indicate that clinicians need to assess, consider, and if relevant, treat impairment of the iUL to effectively manage upper limb rehabilitation after stroke. While the contralateral limb should remain the primary focus of upper limb rehabilitation, this review provides evidence to support the importance of bilateral interventions thereby addressing deficits of both the contralateral upper limb and the iUL [32].

The results of this review validate a change in upper limb vocabulary, and we recommend that the term “less affected” be used when referring to the iUL and the term “more affected” be used when referring to the contralateral upper limb. Health professionals should cease using the terms “unaffected” and “nonaffected” to describe the iUL following stroke, as these terms are misleading at best.

### 4.2. Study Limitations

Heterogeneity between studies is a limitation of this systematic review. Table 1 illustrates the variances of cohort characteristics. Lesion size and location are not documented in this review; however, it is worth noting that while some researchers took this into consideration when recruiting participants, some made no mention of this at all. The time from stroke onset was another notable limitation with the majority of participants recruited in the chronic phase of stroke (≥six months). Handedness was another between-study inconsistency. Right handed cohorts made up the majority of participants across studies with only some studies considering the association between upper limb function and handedness by comparing the iUL to the equivalent upper limb in control groups.

### 4.3. Further Research

Sunderland et al. [7] are the only authors who describe the effect of cognition on the deficits of the iUL. Therefore, further research is required to establish the impact of cognition on performance of the iUL. Further research is also needed to explore the pathophysiological mechanisms underpinning deficits of the iUL following stroke, and the role each hemisphere may play in the particular deficits exhibited.

### 4.4. Pathophysiological Mechanisms

The pathophysiological mechanisms which result in deficits of the iUL are largely unknown. Evidence at this time suggests various hypotheses; however, further research is needed to provide a definitive explanation. A dominant theory suggests that the ipsilesional uncrossed descending corticospinal pathways may play a role in the movement of the iUL [33]. Alternatively, a body of evidence supports the importance of interhemispheric, transcallosal interactions [17, 34–37]. This suggests that activation of the ipsilateral hemisphere during unilateral upper limb movements might be related to excitatory or inhibitory effects in the contralateral hemisphere [35–38].

## 5. Conclusion

This systematic review finds that people who have experienced stroke can have a deficit of the iUL. Therefore, function in the iUL must be considered in rehabilitation to ensure maximum recovery and opportunities for increased independence. The clinical community needs to update the terminology associated with the iUL to acknowledge that it can be adversely affected and that it should not be used as a benchmark for recovery of function in the contralateral upper limb. While the contralateral limb should remain the primary focus of upper limb rehabilitation, this review provides evidence to support the importance of bilateral interventions thereby addressing deficits of both the contralateral upper limb and the iUL.

## Figures and Tables

**Table 1:** Studies which investigated deficits of the iUL following stroke.

Study	*N* = Sn/Hc	Lesioned hemisphere L/R	Baseline assessment	Mean age* Sn/Hc	Hand dominance Sn/Hc	Standardised outcome measure	Result
Studies that used standardised assessments (*n* = 12)							
Jung et al. [2]	72/20	38/34	Acute	55.5/not reported	Right/not reported	Manual Function Test	Improvement of the iUL plateaued at 1 month with recovery incomplete (*P* < 0.05). Shoulder function less affected and recovered faster when compared to hand function.
Noskin et al. [1]	30	12/18	Acute	61.5	Left *n* = 1, right *n* = 29/normative data	Dynamometer, 9HPT	9HPT performance below normative data at each time point (*z* = − 7.1, −3.6, −2.5, and −2.3). Grip strength unaffected. Initial impairment of the iUL correlated to contralateral deficit (*P* = 0.035).
De Groot-Driessen et al. [11]	57/42	24/33	Sub-acute	52.3/52.1	Left *n* = 6, right *n* = 51/left *n* = 3, right *n* = 39	Finger tapping speed from the Amsterdam Neuropsychological Test Battery, the Barthel Index, Frenchay Activities Index, Sickness Impact Profile	Speed of finger tapping was impaired up to 8 weeks after stroke when normative speeds were reached (*P* = 0.02).
Laufer et al. [4]	9/10	5/4	Sub-acute	63.3/62.6	Right/right	JHFT, 9HPT	Reduced speed (*P* < 0.02).
Morris and Van Wijck [12]	56/50	52/54	Sub-acute	67.9/67.8	Left *n* = 3, right *n* = 53/left *n* = 6, right *n* = 44	Action Research Arm Test, 9HPT, Modified Barthel Index	Baseline assessment of iUL was below normative data (no *P* value). Improvement in timed dexterity following bilateral intervention up to 6 weeks.
Sunderland et al. [7] (Sunderland [19])	30/34	15/15	Sub-acute	62.5/65	Left *n* = 2, right *n* = 28/right	JHFT, Williams Doors Test, Apraxia Assessment, Line Cancellation, Judgement of Line Orientation, Token Test Parts I and V, Dynamometer	Reduced speed on dexterity assessment (*P* < 0.01) and impaired grip strength (*P* < 0.001).
Brasil-Neto and De Lima [13]	25/25	11/14	Chronic	58.24/58.6	Right/right	Moving Touch Pressure Test, Box and Block Test, Dynamometer	Motor impairment (*P* < 0.01), decreased sensory discrimination (*P* < 0.01), and impaired grip strength (*P* < 0.05).
Chestnut and Haaland [5]	52/62	31/21	Chronic	63.6/64.6	Right/right	Williams Doors Test, Timed Manual Performance Test	Motor deficits resulting in functional impairment (*P* < 0.001 to *P* < 0.002).
Desrosiers et al. [6]	43/43	14/29	Chronic	71.8/71.8	Right/right	Box and Block Test, Purdue Pegboard, Upper Extremity Performance Evaluation Test for the Elderly	Fine and gross manual dexterity, motor coordination, global performance, and kinaesthesia impaired (*P* < 0.01 to *P* < 0.0001).
Jebsen et al. [9]	27/300	14/13	Chronic	53.65/not reported	Right/not reported	JHFT	Performance below normative data (*P* < 0.05, *P* < 0.01 to *P* < 0.001).
Spaulding et al. [8]	49	22/27	Not reported	66	left *n* = 3, right *n* = 46/normative data	JHFT	Performance below normative data (*P* < 0.001).
Wetter et al. [3]	58/66	34/24	Chronic	64/64.5	Right/right	JHFT	Impaired motor performance (*P* < 0.001).

Studies that used standardised and nonstandardised assessments (*n* = 7)							
Jones et al. [18]	8/20	3/5	Acute	50.1/54.1	Right/right	Snellen Eye Chart, Dynamometer	Impaired movement, speed, and strength (no *P* value).
Mori and Yamadori [20]	100	55/45	Acute	65.8	Right/normative data	Mini-Mental State Exam, Line Cancellation Test, Line Bisection Test	Right hemisphere stroke only demonstrated an instinctive grasp reaction (*P* < 0.005).
Baskett et al. [21]	41/40	20/20	Sub-acute	68.6/71.6	Right/right	Motor Assessment Scale	Right hemisphere stroke only demonstrated sensory-motor deficit (*P* < 0.005).
Yelnik et al. [15]	36/86	18/18	Sub-acute	54/48	Right/right	9HPT	Stroke group performed below control group for all tasks (*P* < 0.05).
McCrea et al. [10]	20/10	13/7	Chronic	60.9/61.0	Left *n* = 3, right *n* = 17/not reported	Dynamometer	Strength and speed of muscle contraction affected (*P* < 0.001).
Nowak et al. [17]	16/8	8/8	Sub-acute	54.5/56	Right/right	Medical Research Council Motor Strength Scale, Modified Rankin Score, Action Research Arm Test, National Institute of Health Stroke Scale	Movement timing, accuracy, and efficiency affected (*P* < 0.01 to *P* < 0.001).
Quaney et al. [22]	10/14	6/4	Chronic	55.5/53.1	Right/right	Pinch Gauge, Box to Block Test	Impaired grip force (*P* ≤ 0.05).

Studies that only used nonstandardised assessments (*n* = 9)							
Lin et al. [23]	26/24	15/11	Sub-acute	63.4/62.3	Right/right	Nonstandardised	Task constraints showed a significant effect on movement variables (*P* < 0.001).
Swinnen et al. [24]	16/16	7/9	Sub-acute	56.3/56.6	Right/right	Nonstandardised	Deficits in coordination (*P* < 0.05 to *P* < 0.01).
Haaland and Harrington [25]	43/32	25/18	Chronic	63/66	Right/right	Nonstandardised	Speed of the left hemisphere group was slower when target size increased (*P* < 0.001).
Kim et al. [26]	10/20	5/5	Chronic	72.4/72.4	Right/right	Nonstandardised	Prolonged movement and dwell time (*P* < 0.001 to *P* < 0.008).
Kwon et al. [27]	34/38	17/19	Not reported	55.3/55.3	Right/right	Nonstandardised	Accuracy (*P* < 0.01 to *P* < 0.0001) and timing deficits after left hemisphere stroke (*P* < 0.0001).
Pohl and Winstein [28], (Pohl et al. [38])	10/10	5/5, 10/10	Chronic	57.1/57.4	Right/right	Nonstandardised	Increased movement time (*P* < 0.02).
Sugarman et al. [29]	11/5	6/5	Chronic	66.05/67.4	Right/right	Nonstandardised	Abnormal pattern of movement, increased movement time, and segmented movement (no *P* value reported).
Yarosh et al. [30]	7/7	4/3	Chronic	56.7/56.6	Left *n* = 1, right *n* = 6/left *n* = 1, right *n* = 6	Nonstandardised	Impaired speed, trajectory, and sequence of muscle activity (*P* < 0.001).

*Average of the left and right hemisphere stroke cohorts.

Sn: stroke cohort.

Hc: healthy controls.

(9HPT): Nine Hole Peg Test.

(JHFT): Jebsen Hand Function Test.

**Table 2:** Example search methodology: Embase from 1974 till present.

Search terms	Number of articles
Stroke and upper limb; arm	2167
Less affected and stroke and upper limb; arm	29
Nonaffected and stroke and upper limb; arm	14
Ipsilateral and stroke and upper limb; arm	85
